# Increased Hs-CRP/adiponectin ratio is associated with increase carotid intima-media thickness

**DOI:** 10.1186/1476-511X-13-120

**Published:** 2014-07-29

**Authors:** Huocheng Liao, Zhiming Li, Dongdan Zheng, Jianping Liu, Yan Liu, Chun Xiao, Hongguang Wang

**Affiliations:** 1 Department of Cardiology, the 3rd People’s Hospital, Huizhou, China; 2 Department of Cardiology, Huizhou Municipal Central Hospital, 41st Eling North RD, Huicheng District, Huizhou, China; 3 Department of Cardiology, The First Affiliated Hospital of Sun Yat-sen University, Guangzhou, China; 4 Internal Medicine-Cardiovascular Department, Dongying People’s Hospital of Shandong Province, NO.137, Nanyi Road, Dongying, Shangdong Province, China

**Keywords:** High sensitivity C-reactive protein, Adiponectin, Atherosclerosis

## Abstract

**Background:**

High sensitivity C-reactive protein (Hs-CRP) and adiponectin (APN) are two critical cytokines and exert inverse effects on atherosclerosis initiation and progression. The purpose of our study was to investigate the value of Hs-CRP and ANP ratio (Hs-CRP/APN ratio) on evaluating atherosclerosis progression.

**Method:**

One hundred sixty consecutive participants underwent carotid intima-media thickness (CIMT) measured by ultrasound were enrolled and drawn fasting blood samples for plasma levels Hs-CRP and APN, serum levels of lipid profiles and fasting blood glucose evaluation. Other anthropometrics and clinical status were collected by questionnaire. All participants were divided into 4 groups according to the baseline Hs-CRP/APN ratio and underwent CIMT measurement every 6 months. CIMT increment and composite cardiovascular endpoints were compared after 24 months’ follow-up.

**Results:**

At baseline, body mass index (BMI), smoking, diabetic mellitus, usage of statins, Hs-CRP and APN independently correlated with Hs-CRP/APN ratio as analyzed by spearman rank correlation. Smoking, serum level of LDL-C, plasma level of Hs-CRP and Hs-CRP/APN ratio were positively correlated with CIMT while usage of statins and plasma level of APN were negatively correlated with CIMT as analyzed by multiple linear regression analysis. After 24 months’ follow-up, the progression of CIMT was the most prominent in the fourth quartile of baseline Hs-CRP/APN ratio. In addition, the incidence of composite cardiovascular endpoint was also higher in the fourth quartile as compared to the other 3 lower quartiles.

**Conclusion:**

Hs-CRP/APN ratio was a useful predictor to discriminate subjects who were at increased risk of atherosclerosis progression.

## Introduction

In the past decades, accumulating evidence from basic and clinical studies consistently demonstrate that inflammation plays pivotal and complicated roles in the initiation and progression of atherosclerosis and atherosclerotic cardiovascular diseases [1-3]. Notably, increased serum level of inflammatory cytokines portends a higher incidence of cardiovascular events such as myocardial infarction and ischemic stroke [4,5]. Therefore, guidelines have recommended that inflammatory cytokines such as high sensitivity C-reactive protein (Hs-CRP) should be integrated into the algorithm of cardiovascular risk evaluation [1,6]. Knowingly, Hs-CRP is a non-specific inflammatory cytokine, and by means of increasing inflammatory cells infiltration, augmenting oxidative stress, impairing endothelial function as well as decreasing nitric oxide (NO) production, Hs-CRP accelerates the formation and disruption of atherosclerotic plaque [7-9]. On the contrary, adiponectin (APN) is a cardio-protective cytokine due to its capability to inhibiting inflammatory cytokines such as interleukin-6, TNF-α and Hs-CRP expressions, down-regulating monocytes chemoattractant proein-1 (MCP-1) expression, increasing NO generation as well as enhancing myocardial blood perfusion [10-13].

Carotid intima-media thickness (CIMT), an early and sensitive indicator for subclinical atherosclerosis, is significantly associated with plasma levels of Hs-CRP and APN as reported by previous studies [14,15]. With respect to the aforementioned mechanisms by which Hs-CRP and APN exert on cardiovascular system, we hypothesized that Hs-CRP and APN ratio (Hs-CRP/APN ratio) might be useful as an indicator to help us discriminate the status of atherosclerosis. Thus that, we conducted a pilot study to investigate the relationship between Hs-CRP/APN ratio and CIMT in population without overt cardiovascular diseases, and then we consecutively measured CIMT in the follow-up 24 months so as to determine the value of Hs-CRP/APN ratio in predicting CIMT progression and future cardiovascular events.

## Methods

### Study population enrollment

One hundred and sixty consecutive subjects were randomly enrolled from January 2011 to January 2012, and all subjects were underwent CIMT measurement by duplex ultrasound. Written informed consent was obtained and the ethics committee of the 3rd People’s Hospital of Huizhou approved present study. Subjects who initially without overt cardiovascular diseases, which defined as angina pectoris, previous myocardial infarction, transient ischemia attack or ischemic stroke and peripheral artery diseases, were ruled in, and those with a previous history of atherosclerotic cardiovascular diseases or baseline level of Hs-CRP >10 mg/L, which indicated intensively infectious diseases or collagen diseases or potential tumors, were ruled out.

### Demographic and clinical characteristics collection

Demographic and clinical characteristics such as age, gender, body mass index (BMI), smoking status, hypertension or current anti-hypertension therapy, dyslipidemia or use of lipid-lowering medications and diabetic mellitus were obtained by the means of computerized questionnaire.

### Laboratory examination

Fasting blood samples were drawn from each participant and transferred on ice to center laboratory for analysis. An IMMAGE automatic immunoassay system (Beckmann-Coulter) was used to examine plasma level of Hs-CRP. Intra-assay variability coefficient lied between 3.5-5.0%, and inter-assay variability coefficient between 4.0-7.5%, and limited detection level was 0.2 mg/L. APN was measured by a high sensitivity Human adiponectin ELISA kit (BioCat GmbH) with intra-assay variability coefficient lied between 4.0-5.5% and inter-assay variability coefficient between 5.0-7.5%. Triglyceride (TG), total cholesterol (TC), low density lipoprotein-cholesterol (LDL-C), high density lipoprotein-cholesterol (HDL-C) and fasting blood glucose were also measured by Automatic Biochemistry Analyzer (Beckman coulter UniCel DxC 800 Synchron).

### Carotid intima-media thickness measurement

Ultrasonic examination was performed with an 8.5 MHz linear array transducer. The initial and follow-up measurements were performed by two investigators who were blinded to the characteristics of all participants. Common carotid artery (one milimeter below the carotid artery bifurcation) was chosen to evaluate CIMT on both sides. The average inter-observers reliability coefficient was 0.95 (95% confidence interval (CI) 0.93 to 0.96, p < 0.05) and the intra-observer reliability coefficient 0.93 (95% CI 0.91 to 0.94, p < 0.05). The values of CIMT were averaged by the left and right CIMT values. The absolute annual change of CIMT was determined by the following formula: (final value minus baseline value) divided by the total follow up years.

### Clinical endpoints

All subjects were followed up for the occurrence of first cardiovascular events, including myocardial infarction, ischemic stroke, and cardiovascular diseases related death. Total follow-up lasted for 24 months.

### Statistical analysis

All analyses were performed by SPSS16.0 package. All values were expressed as mean ± SD or percentage. Correlation between Hs-CRP/APN ratio with demographic and clinical characteristics were evaluated by Pearson correlation or Spearman rank correlation as appropriate. Independent correlation between Hs-CRP/APN ratio and other variables with CIMT were determined by multiple linear regressions. Subjects were divided into four groups according to the baseline quartiles of Hs-CRP/APN ratio. Comparison of CIMT value of each quartile at the same time point, and comparison of CIMT increment of each quartile during follow-up were operated by one-way ANOVA. A p value < 0.05 was considered to be significant difference.

## Results

### Baseline characteristics and relationship between Hs-CRP/APN ratio and other variables

As shown in Table 1, the age of participants was 55.3 ± 4.8 years and 45.0% of participants were female. Approximately 58.1% were cigarette smoker, BMI was 26.5 ± 4.1 Kg/m^2^, 45.0% with diabetic mellitus, 53.1% with hypertension and nearly 51.9% with anti-hypertension therapy, current statins usage were 49.3%. Plasma levels of Hs-CRP and APN were 6.9 ± 1.8 mg/L and 10.8 ± 2.2 μg/L, respectively, and the Hs-CRP/APN ratio was 0.6 ± 0.2. Correlation between Hs-CRP/APN ratio and other variables was shown in Table 1. For all the variables, BMI, smoking, diabetic mellitus, usage of statins, and plasma levels of Hs-CRP and APN significantly correlated with Hs-CRP/APN ratio (all p values < 0.05).

**Table 1:** Baseline characteristics and relationship between Hs-CRP/APN ratio and other variables (n = 160)

**Variables**	**Mean ± SD or percentage**	**r**	**P**
Age (years)	55.3 ± 4.8	0.21	0.34
Female (%)	72 (45.0)	0.18	0.65
BMI (Kg/m^2^)	26.5 ± 4.1	0.43	<0.05
Smoking (%)	93 (58.1)	0.72	<0.05
Diabetic mellitus (%)	72 (45.0)	0.34	<0.05
Hypertension (%)	85 (53.1)	0.58	0.15
TG (mmol/L)	1.5 ± 0.5	0.33	0.13
TC (mmol/L)	5.6 ± 0.4	0.26	0.25
HDL-C (mmol/L)	1.6 ± 0.7	0.25	0.16
LDL-C (mmol/L)	4.3 ± 0.8	0.35	0.08
Usage of statins (%)	79 (49.3)	−0.67	<0.05
Anti-hypertension	83 (51.9)	−0.24	0.78
Hs-CRP (mg/L)	6.9 ± 1.8	0.79	<0.001
APN (μg/L)	10.8 ± 2.2	−0.83	<0.001
Hs-CRP/APN ratio	0.6 ± 0.2		

Spearman’s rank correlation coefficient (r) is shown.

### Relationship between CIMT and Hs-CRP/APN ratio and other variables

Relationship between CIMT and Hs-CRP/APN ratio and other variables was shown in Table 2. Notably, CIMT significantly correlated with smoking, serum level of LDL-C, usage of statins, plasma levels of Hs-CRP and APN, and Hs-CRP/APN ratio. Specifically, usage of statins and plasma level of APN were negatively correlated with CIMT (p < 0.05), and other variables including smoking, serum level of LDL-C, plasma level of Hs-CRP and Hs-CRP/APN ratio were positively correlated with CIMT (p < 0.05).

**Table 2:** Relationship between CIMT and Hs-CRP/APN ratio and other variables

**Characteristics**	**β**	**P**
Age	0.24	0.36
Female	0.43	0.15
BMI	0.23	0.11
Smoking	0.18	0.04
Diabetic mellitus	0.27	0.08
Hypertension	0.29	0.16
TG	0.15	0.57
TC	0.27	0.64
HDL-C	−0.22	0.12
LDL-C	0.25	0.03
Usage of statins	−0.33	0.01
Anti-hypertension	0.33	0.28
Hs-CRP	0.46	0.01
APN	-.031	0.01
Hs-CRP/APN ratio	0.53	0.02

β standard regression coefficient.

### Comparison of CIMT within quartiles of Hs-CRP/APN ratio

According to baseline Hs-CRP/APN ratio, participants were divided into four quartiles. Hs-CRP/APN ratio from the first to the fourth quartile was 0.10 ± 0.03, 0.34 ± 0.06, 0.50 ± 0.10 and 0.68 ± 0.13, respectively. As presented in Table 3, CIMT was significantly different between each quartile at the same time point and the increment of CIMT in the fourth quartile was the greatest when compared to the other 3 groups.

**Table 3:** Comparison of CIMT within quartiles of Hs-CRP/APN ratio

**CIMT (mm)**	**1**^**st **^**(n = 40)**	**2**^**nd **^**(n = 40)**	**3**^**rd **^**(n = 40)**	**4**^**th **^**(n = 40)**	**P**
Baseline	0.65 ± 0.14	0.67 ± 0.11	0.70 ± 0.17	0.72 ± 0.15^#^	< 0.05
6 months	0.66 ± 0.13	0.69 ± 0.15	0.72 ± 0.12	0.73 ± 0.14^#^	< 0.05
12 months	0.68 ± 0.10	0.72 ± 0.11	0.74 ± 0.14	0.76 ± 0.19^#^	< 0.05
18 months	0.70 ± 0.11	0.74 ± 0.20	0.77 ± 0.18	0.81 ± 0.18^#^	< 0.05
24 months	0.71 ± 0.14	0.77 ± 0.19*	0.79 ± 0.16*	0.86 ± 0.20*^#^	< 0.001
P	0.145	< 0.05	< 0.05	< 0.001	

Denote: 1^st^ = the first quartile, 2^nd^ = the second quartile, 3^rd^ = the third quartile, and 4^th^ = the fourth quartile; *P < 0.05, versus baseline level of CIMT at the same quartile, ^#^P < 0.05, versus the first quartile of CIMT at the same time point.

### Comparison of clinical events

During the 24 months’ follow-up, there were totally 4 cases of myocardial infarction and 6 cases of ischemic stroke occurred. Notably, the incidence of composite endpoint was higher in the fourth quartile (6 cases) than the other 3 groups as shown in Table 4. In the fourth quartile, the first episode of clinical event happened in the first ten month, while it was in the eighteenth month when the first clinical event occurred in the first quartile.

**Table 4:** Comparison of clinical events

**Cardiovascular events**	**1**^**st **^**(n = 40)**	**2**^**nd **^**(n = 40)**	**3**^**rd **^**(n = 40)**	**4**^**th **^**(n = 40)**	**P**
MI	0	0	1	3	NS
Stroke	0	1	2	3	NS
Death	0	0	0	0	NS

Denote: MI = myocardial infarction, NS = no significant.

## Discussion

Our present study shows that in participants without overt atherosclerotic cardiovascular diseases, increased baseline Hs-CRP/APN ratio portends a higher risk of CIMT progression and a higher incidence of cardiovascular events during 24 months follow-up. Future study is imperative in investigating whether decreasing Hs-CRP/APN ratio could result in better cardiovascular outcomes.

Previously, a substantial number of studies have reported the association between cardiovascular risk factors and plasma levels of Hs-CRP and APN. For example, BMI was reported positively correlated with Hs-CRP level while negatively with APN level [15,16], which therefore might partially explain the positive relationship between BMI and Hs-CRP/APN ratio in our current study. Smoking is a crucial risk factor contributing to continuous systemic inflammation due to its effects on impairing endothelial function and promoting vascular inflammation [17]. Commonly, in population with long-term cigarette smoking, the plasma level of Hs-CRP was increased therefore resulting in the increased Hs-CRP/APN ratio. Accordingly, most of the complications of diabetes mellitus resulted from endothelial dysfunction and activation [18,19], which characterized by reduced NO production and inflammatory cytokines up-regulation and subsequently leaded to plasma levels of Hs-CRP increase and APN decrease. Previously, many primary and secondary studies have revealed the pleiotropic effects of statins, which was largely ascribed to the reduction of Hs-CRP level and increase NO production [20,21]. Therefore, it was no wonder that statins was negatively correlated with Hs-CRP/APN ratio. However, In the future, to assess whether modifying above factors could result in a favorable Hs-CRP/APN ratio is imperative.

Carotid intima-media thickness (CIMT) has been broadly considered an important parameter to identify an early status of atherosclerosis, and measure of CIMT could facilitate physicians to discriminate those who are at increased risk of cardiovascular events [22,23]. In current study, smoking, serum level of LDL-C, usage of statins, plasma levels of Hs-CRP and APN, and Hs-CRP/APN ratio were found significantly correlated with CIMT as evaluated by multiple linear regression analysis. Based on previous studies [17-21], it was reasonable to explain the relationship between CIMT and cardiovascular risk factors such as smoking and LDL-C. Nevertheless, this was the first time we reported that Hs-CRP/APN ratio was also significantly associated with CIMT. Since plasma level of Hs-CRP and APN were affected by many conditions as reported above, it was not possible for us to conclude that Hs-CRP/APN ratio was an independent risk factor for CIMT progression. However, because Hs-CRP/APN ratio could reflect a broader spectrum of pathophysiological situation in terms of both pro-inflammation and anti-inflammation, we believed that incorporating Hs-CRP/APN ratio into cardiovascular risk assessment was useful and helpful for improving the capacity of risk discrimination. Outcome from our follow-up study was also supported for our reasoning. All participants were follow-up for 24 months and underwent serials CIMT measurement every 6 months. Results showed that the progression of CIMT was more significant in the fourth quartile of Hs-CRP/APN ratio. Additionally, the incidence of composite outcomes was also higher in the fourth quartile than that of the other 3 lower quartiles, indicating that baseline Hs-CRP/APN ratio was a useful parameter for helping us to identify those who were at increased risk of cardiovascular events.

Finally, there were some limitations of current study. First of all, the sample size was not large enough. Secondly, because current study was an observational study, one could not make definite causal relationship between Hs-CRP/APN ratio and CIMT progression and cardiovascular events. Additionally, there were some inhere biases of observational studies which physicians should bear in mind when interpreted current study.

## Conclusion

In conclusion, according to our current study, Hs-CRP/APN ratio was a useful predictor for atherosclerosis progression and clinical events. Because Hs-CRP/APN ratio highly correlated with BMI, smoking, diabetic mellitus, usage of statins, and meanwhile baseline Hs-CRP/APN ratio significantly correlated with CIMT progression and the incidence of clinical events, we considered that further study should be focused on lifestyle modification, glucose control and usage of statins so as to find out whether tough control of these risks factors and more intensive therapy with statins could improve the Hs-CRP/APN ratio which finally results in better clinical outcomes.

## Competing interests

The authors declare that they have no competing interests.

## Authors’ contributions

HL, ZL, JL and YL performed this study, DZ performed statistic analyses, and CX and HW designed this study, and HL and ZL wrote this article. All authors read and approved the final manuscript.

## Authors’ information

Huocheng Liao and Zhiming Li co-first authors.
